# Sustained oxygenation improvement after first prone positioning is associated with liberation from mechanical ventilation and mortality in critically ill COVID-19 patients: a cohort study

**DOI:** 10.1186/s13613-021-00853-1

**Published:** 2021-04-26

**Authors:** Gaetano Scaramuzzo, Lorenzo Gamberini, Tommaso Tonetti, Gianluca Zani, Irene Ottaviani, Carlo Alberto Mazzoli, Chiara Capozzi, Emanuela Giampalma, Maria Letizia Bacchi Reggiani, Elisabetta Bertellini, Andrea Castelli, Irene Cavalli, Davide Colombo, Federico Crimaldi, Federica Damiani, Maurizio Fusari, Emiliano Gamberini, Giovanni Gordini, Cristiana Laici, Maria Concetta Lanza, Mirco Leo, Andrea Marudi, Giuseppe Nardi, Raffaella Papa, Antonella Potalivo, Emanuele Russo, Stefania Taddei, Guglielmo Consales, Iacopo Cappellini, Vito Marco Ranieri, Carlo Alberto Volta, Claude Guerin, Savino Spadaro, The ICU-RER COVID-19 Collaboration, Marco Tartaglione, Valentina Chiarini, Virginia Buldini, Carlo Coniglio, Federico Moro, Clara Barbalace, Mario Citino, Nicola Cilloni, Lorenzo Giuntoli, Angela Bellocchio, Emanuele Matteo, Giacinto Pizzilli, Antonio Siniscalchi, Chiara Tartivita, Francesco Matteo, Annalisa Marchio, Igor Bacchilega, Laura Bernabé, Sonia Guarino, Elena Mosconi, Luca Bissoni, Lorenzo Viola, Tommaso Meconi, Vittorio Pavoni, Aline Pagni, Patrizia Pompa Cleta, Marco Cavagnino, Anna Malfatto, Angelina Adduci, Silvia Pareschi, Gabriele Melegari, Jessica Maccieri, Elisa Marinangeli, Fabrizio Racca, Marco Verri, Giulia Falò, Elisabetta Marangoni, Francesco Boni, Giulia Felloni, Federico Domenico Baccarini, Marina Terzitta, Stefano Maitan, Filippo Becherucci, Maddalena Parise, Francesca Masoni, Michele Imbriani, Paolo Orlandi, Francesco Monetti, Giorgia Dalpiaz, Rita Golfieri, Federica Ciccarese, Antonio Poerio, Francesco Muratore, Fabio Ferrari, Martina Mughetti, Loredana Franchini, Ersenad Neziri, Marco Miceli, Maria Teresa Minguzzi, Lorenzo Mellini, Sara Piciucchi, Maurizio Bartolucci

**Affiliations:** 1 Department of Translational Medicine and for Romagna, University of Ferrara & Azienda Ospedaliero-Universitaria S. Anna, Via Aldo Moro, 8 Cona, 44121 Ferrara, Italy; 2 Department of Anaesthesia, Intensive Care and Prehospital Emergency, Ospedale Maggiore Carlo Alberto Pizzardi, Bologna, Italy; 3 Alma Mater Studiorum, Dipartimento di Scienze Mediche e Chirurgiche, Anesthesia and Intensive Care Medicine, Policlinico di Sant’Orsola, Università di Bologna, Bologna, Italy; 4 Department of Anesthesia and Intensive Care, Santa Maria Delle Croci Hospital, Ravenna, Italy; 5 Cardio-Anesthesiology Unit, Cardio-Thoracic-Vascular Department, S.Orsola Hospital, University of Bologna, Bologna, Italy; 6 Radiology Department, M. Bufalini Hospital, Cesena, Italy; 7 Department of Clinical, Integrated and Experimental Medicine (DIMES), Statistical Service, Alma Mater University, S. Orsola-Malpighi Hospital Bologna, Bologna, Italy; 8 Department of Anaesthesiology, University Hospital of Modena, Via del Pozzo 71, 41100 Modena, Italy; 9 Anaesthesia and Intensive Care Department, SS. Trinità Hospital, ASL, Novara, Italy; 10 Translational Medicine Department, Eastern Piedmont University, Novara, Italy; 11 Eastern Piedmont University, Novara, Italy; 12 Department of Anaesthesia, Intensive Care and Pain Therapy, Imola Hospital, Imola, Italy; 13 Anaesthesia and Intensive Care Unit, M. Bufalini Hospital, Cesena, Italy; 14 Anesthesia and Intensive Care Unit of Transplant, Department of Organ Failures and Transplants, Azienda Ospedaliero-Universitaria Di Bologna (IRCCS), Bologna, Italy; 15 Department of Anesthesia and Intensive Care, G.B. Morgagni-Pierantoni Hospital, Forlì, Italy; 16 Department of Anaesthesia and Intensive Care, Azienda Ospedaliera SS. Antonio E Biagio E Cesare Arrigo, Alessandria, Italy; 17 Department of Anaesthesia and Intensive Care, Infermi Hospital, Rimini, Italy; 18 Anaesthesia and Intensive Care Unit, Santa Maria Annunziata Hospital, Firenze, Italy; 19 Anaesthesia and Intensive Care Unit, Bentivoglio Hospital, Bentivoglio, Bologna, Italy; 20 Department of Critical Care Section of Anesthesiology and Intensive Care, Azienda USL Toscana Centro, Prato, Italy; 21 Médecine Intensive-Réanimation Groupement Hospitalier Edouard Herriot, Université de Lyon Faculté de Médecine Lyon-Est, Lyon, Institut Mondor de Recherches Biomédicales, Créteil, France

**Keywords:** COVID19, Prone positioning, Ventilatory free days, ICU

## Abstract

**Background:**

Prone positioning (PP) has been used to improve oxygenation in patients affected by the SARS-CoV-2 disease (COVID-19). Several mechanisms, including lung recruitment and better lung ventilation/perfusion matching, make a relevant rational for using PP. However, not all patients maintain the oxygenation improvement after returning to supine position. Nevertheless, no evidence exists that a sustained oxygenation response after PP is associated to outcome in mechanically ventilated COVID-19 patients. We analyzed data from 191 patients affected by COVID-19-related acute respiratory distress syndrome undergoing PP for clinical reasons. Clinical history, severity scores and respiratory mechanics were analyzed. Patients were classified as responders (≥ median PaO_2_/FiO_2_ variation) or non-responders (< median PaO_2_/FiO_2_ variation) based on the PaO_2_/FiO_2_ percentage change between pre-proning and 1 to 3 h after re-supination in the first prone positioning session. Differences among the groups in physiological variables, complication rates and outcome were evaluated. A competing risk regression analysis was conducted to evaluate if PaO_2_/FiO_2_ response after the first pronation cycle was associated to liberation from mechanical ventilation.

**Results:**

The median PaO_2_/FiO_2_ variation after the first PP cycle was 49 [19–100%] and no differences were found in demographics, comorbidities, ventilatory treatment and PaO_2_/FiO_2_ before PP between responders (96/191) and non-responders (95/191). Despite no differences in ICU length of stay, non-responders had a higher rate of tracheostomy (70.5% vs 47.9, *P* = 0.008) and mortality (53.7% vs 33.3%, *P* = 0.006), as compared to responders. Moreover, oxygenation response after the first PP was independently associated to liberation from mechanical ventilation at 28 days and was increasingly higher being higher the oxygenation response to PP.

**Conclusions:**

Sustained oxygenation improvement after first PP session is independently associated to improved survival and reduced duration of mechanical ventilation in critically ill COVID-19 patients.

**Supplementary Information:**

The online version contains supplementary material available at 10.1186/s13613-021-00853-1.

## Background

To date, SARS-CoV-2 has infected more that 132 million people of whom more than 2.8 million died worldwide [1]. When the case fatality ratio is not homogeneous across countries, probably depending on the number of tests done, the mortality in patients admitted to the Intensive Care Unit (ICU) is higher than 40% [2] and can reach 73% for patients requiring both mechanical ventilation and dialysis [3]. The pneumonia caused by SARS-CoV-2 (COVID-19), indeed, can lead to severe Acute Respiratory Distress Syndrome (ARDS) requiring invasive mechanical ventilation. COVID-19-related ARDS is associated to a severe impairment of lung ventilation/perfusion $$\left( {{\raise0.7ex\hbox{${\dot{V}_{A} }$} \!\mathord{\left/ {\vphantom {{\dot{V}_{A} } {\dot{Q}}}}\right.\kern-\nulldelimiterspace} \!\lower0.7ex\hbox{${\dot{Q}}$}}} \right)$$ matching, resulting from a defect of hypoxic pulmonary vasoconstriction and presence of thrombi in the pulmonary microcirculation, leading to high intra-pulmonary shunt and dead space, respectively [4, 5]. Early prone positioning (PP) has been used in critically ill patients affected by the COVID-19, both in patients receiving invasive mechanical ventilation [6] and in those spontaneously breathing [7]. In non-COVID-19 severe ARDS, PP has an established role [8], as it can improve oxygenation and survival as compared to supine position [9]. The mechanisms by which PP improves oxygenation include the recruitment of atelectatic dorsal lung areas and the redistribution of lung ventilation toward still well perfused areas [10, 11]. Moreover, PP may reduce the lung stress and strain associated with mechanical ventilation, thus decreasing the risk of ventilator-induced lung injury [12].

Though the improvement of oxygenation resulting from PP may be dramatic in some patients, it is not observed in every one of them. The COVID-19-related hypoxemia is, indeed, caused by a combination of several factors, which affect $${\raise0.7ex\hbox{${\dot{V}_{A} }$} \!\mathord{\left/ {\vphantom {{\dot{V}_{A} } {\dot{Q}}}}\right.\kern-\nulldelimiterspace} \!\lower0.7ex\hbox{${\dot{Q}}$}}$$ in different ways, making COVID-19 ARDS patients potentially responding differently to PP in terms of oxygenation. Moreover, the relationship between oxygenation response to PP and survival is still an open question, since it has been found either marginally [13] or not associated [14] with survival in patients with non-COVID-19 ARDS. Several evidences exists that in many proned COVID-19 patients, the oxygenation improvement determined by PP is not sustained after re-supination [7, 15, 16]. To date, no evidence exists that a sustained improvement of oxygenation after resupination is associated to patient’s outcome. Therefore, we aimed at further explore whether, in mechanically ventilated COVID-19 patients undergoing PP, an oxygenation improvement to PP sustained after resupination would be associated to patient outcome. To investigate this, we performed a secondary analysis on consecutive mechanically ventilated ARDS COVID-19 patients admitted to 16 Italian ICUs and undergoing PP for clinical decision. We analyzed if the oxygenation variation after the first PP session, as compared to the pre-PP state, could be associated to ICU ventilatory-free days (VFD), ICU mortality and likelihood of liberation from mechanical ventilation assessed at 28 days after ICU admission.

## Methods

This is a secondary analysis on patients enrolled in a previous prospective study [17] conducted in 15 ICUs from Italian hospitals between February 22 and May 4, 2020. The data from another ICU obtained after the publication of the first paper were included in present report.

The study was approved by the Institutional Review Board of the study coordinator center (Maggiore Hospital, Bologna, Italy, approval number: 273/2020/OSS/AUSLBO) and by each institutional review committee of the participating hospitals. Informed consent was partially waived according to the approval of the local Ethics committee and analysis was conducted on anonymized individual data. The study was registered in ClinicalTrials.gov (NCT04411459).

### Inclusion and exclusion criteria

To be included in the present study patients should be tested positive for SARS-CoV-2 infection (confirmed by real-time reverse transcription-polymerase chain reaction assays), older than 18 years, receive invasive mechanical ventilation, undergo at least one PP session for which PaO_2_/FiO_2_ and driving pressure (DP) data were available (Additional file 1: Figure S1) and fulfill the criteria for ARDS, according to the Berlin definition [8]. The single non-inclusion criterion was the use of non-invasive ventilation (NIV) during the entire clinical course.

### Data collection and mechanical measurements

Baseline data and patient’s history were collected using an electronic case report form developed by YGHEA, CRO division of Ecol Studio SPA (Bologna Operational Headquarters, Bologna, Italy) and hosted by Actide Nubilaria (Novara, Italy). Collected data included demographic data, clinical symptoms or signs at presentation, underlying comorbidities, laboratory data, chest radiologic reports, respiratory parameters before the intubation and ventilator settings during the first 5 days of mechanical ventilation [e.g., positive end-expiratory pressure (PEEP), plateau pressure (Pplat), static compliance (*C*_RS_), PaO_2_/FiO_2_ ratio], and clinical outcomes. The PaO_2_/FiO_2_ and DP were recorded within 3 h before PP (T1) and from 1 to 3 h after re-supination (T2). DP was computed as Pplat minus total PEEP. DP (DPdiff) and PaO_2_/FiO_2_ difference (Pfdiff) were calculated as the difference in DP and in PaO_2_/FiO_2_, respectively, between T1 and T2 (T2 minus T1). Both measurements were taken; therefore, in supine position, the duration of the PP session was recorded.

### Definitions

Mechanical ventilation was considered invasive if delivered through an endotracheal tube or a tracheostomy cannula. The duration of invasive mechanical ventilation was defined as the time elapsed from intubation to successful extubation or successful permanent disconnection from mechanical ventilation for tracheostomized patients. This latter was considered effective if sustained 24 h per day. Extubation failure was defined as the need for reintubation within 48 h from extubation. VFDs at 28 days were defined as 28 minus the number of days elapsed from the last successful extubation in intubated patients, whether or not NIV was used after extubation. VFDs were defined as zero in patients who died during the 28 days regardless of their extubation status. In tracheostomized patients, intermittent disconnections were not counted and VFDs were defined as 28 minus the number of days from the last successful sustained disconnection from mechanical ventilation. A cut-off of 40 ml/cmH_2_O was chosen for discriminating between higher and lower C_RS_ as previously suggested [8]. To define responders and non-responders to PP, we evaluated the distribution of PaO_2_/FiO_2_ response, calculate as percentage of PaO_2_/FiO_2_ change in T2 as compared to T1. Responders were defined as the patients in which the P/F increase in T2 was ≥ of the median population response, while non-responders were those with a percentage P/F change in T2 < median response of the general population.

### Statistical analysis

Continuous variables were expressed as median and first-to-third interquartile range (IQR), unless otherwise stated, while categorical variables were expressed as counts and percentage, and compared using Mann–Whitney *U* test and Chi-square test, respectively. The differences of PaO_2_/FiO_2_ ratio and driving pressure before and after pronation between the responders and non-responders [18] groups were analyzed with a general linear model for repeated measures. Correlation between variables was tested using the Pearson R test. Ventilator-free days were expressed as mean ± SD, as suggested by Yehya et al. [19]. The liberation from mechanical ventilation at 28 days after intubation (D0) was analyzed by performing a competing risks regression model on data, according to the method of Fine and Gray [20], with the event death being the competing risk. The response to prone positioning was analyzed as ordinal variable, grouping patients into 4 classes of response based on the quartiles of PaO_2_/FiO_2_ response to first PP distribution. Model building was performed by means of a variable selection based on an initial screening using univariate analysis with a *P* value < 0.2 criterion, then a stepwise selection with entry criterion at *P* value = 0.05 and stay criterion at *P* value = 0.1. Estimates of coefficients in the model are reported as sub-hazard ratios (95% confidence intervals (CI). All *P* values refers to two-tailed tests of significance and *P* < 0.05 was deemed as the statistically significant threshold. Data were analyzed using SPSS Statistics 26 (IBM SPSS Statistics for Windows, Version 26.0. Armonk, NY: IBM Corp.) and Stata/IC 16 (College Station, Texas, USA). Post-hoc power was calculated on the primary outcome of the study (VFDs) using G*Power 3.1.9.4.

## Results

### Sample selection

Of the 470 patients in the original database, 313 (66.6%) underwent PP during ICU stay and were screened for eligibility. Complete data on respiratory variables before and after prone positioning were available for 191/313 (61%) patients, who were subsequently considered in the current analysis.

### Main characteristics of the sample

The main characteristics of the population are summarized in Table 1. Age was 66 years [59–72], 152 patients (79.6%) were males, SAPS II and SOFA scores at admission were 38 [30–45] and 5 [3–7], respectively. Hypertension was the most common comorbidity (104/191, 54.5%). NIV, continuous positive airway pressure (CPAP) or high flow nasal oxygen (HFNO) were used in most of the patients before intubation and most of them underwent NIV or CPAP for more than 24 h at the time of intubation. PaO_2_/FiO_2_ observed before intubation was 94 [76–112] mmHg.

**Table 1:** Demographic data and clinical characteristics

	Total population (*n* = 191)	Responders (*n* = 96)	Non responders (*n* = 95)	*P*
Age—yr	66 [59–72]	65 [59–72]	68 [59–72]	0.25
Sex—male—no (%)	152 (79.6)	75 (78)	77 (81)	0.72
BMI	28 [26–31]	28 [25–31]	28 [26–31]	0.35
SAPS II score	38 [30–45]	38[30–46]	38[30–44]	0.81
SOFA score	5 [3–7]	4 [3–6]	5 [3–7]	0.31
Hypertension—no (%)	104 (54.5)	50 (52)	54 (57)	0.56
Chronic ischemic heart disease—no (%)	20 (10.5)	8 (8)	12 (13)	0.36
Chronic kidney disease—no (%)	12 (6.3)	5 (5.2)	7 (7.4)	0.57
Diabetes—no (%)	46 (24.1)	22 (22.9)	24 (25.3)	0.74
Chronic liver disease (MELD > 10)—*n* (%)	3 (1.6)	1 (1)	2 (2.1)	0.62
COPD—no (%)	14 (7.3)	8 (8.3)	6 (6.3)	0.39
HFNO before intubation—no (%)—183	20 (10.9)	13 (13.5)	7 (7.4)	0.24
CPAP/NIV before intubation—no (%)	127 (70.4)	60 (62.5)	67 (70.5)	0.20
PaO_2_/FiO_2_ before intubation—mmHg	94 [76–112]	90 [70–113]	96 [80–110]	0.50
Tidal volume set—ml/kg IBW	7.2 [6.6–7.8]	7.3[6.5–8.1]	7.1[6.6–7.6]	0.48
PEEP set—cmH_2_O	12 [10–14]	12 [10–15]	12 [10–14]	0.80
Highest Pplat^a^—cmH_2_O	25 [23–28]	25 [22–28]	26 [24–29]	**0.04**
Lowest C_RS_ supine^a^—ml/cmH_2_O	35 [29–41]	37 [30–43]	33 [27–40]	**0.005**
Lowest PaO_2_/FiO_2_^a^—mmHg	90 [69—113]	89 [67—114]	90 [70—110]	0.87
Class 3—P/F200—300 (%)	0 (0)	0 (0)	0 (0)	
Class 2—P/F 100—200 (%)	72 (38.8)	40 (41.7)	32 (33.7)	
Class 1—P/F < 100 (%)	119 (62.3)	56 (58.3)	63 (66.3)	
Duration of CPAP/NIV trial before intubation	***(n = 127)***	***(n = 60)***	***(n = 67)***	0.23
< 12 h—no (%)	26 (20.5)	8 (13.3)	18 (26.9)	
12—24 h—no (%)	31 (24.4)	16 (26.7)	15 (22.4)	
24—48 h—no (%)	22 (17.3)	13 (21.7)	9 (13.4)	
> 48 h—no (%)	48 (37.8)	23 (38.3)	25 (37.3)	

Data expressed as median [IQR] or counts (% in group). Comparisons were performed using Mann–Whitney *U* test or Chi-square test. Significant *P* values are highlighted in bold

*BMI*  body mass index, *SAPS*  simplified acute physiology score, *SOFA*  sequential organ failure assessment score, *COPD*  chronic obstructive pulmonary disease, *CPAP*  continuous positive airway pressure, *MELD*  model for end-stage liver disease, *NIV*  non-invasive ventilation, *PaO*_*2*_  arterial oxygen partial pressure, *FiO*_*2*_  inspired fraction of oxygen, *IBW*  Ideal Body Weight, *PEEP*  positive end expiratory pressure, *Pplat*  plateau pressure, *CRS*  respiratory system compliance, *HFNO*  high flow nasal oxygen, *PP*  prone position, *DP*  driving pressure

^a^During the first 5 days of ICU stay

### Responders and non-responders comparability

The median PaO_2_/FiO_2_ improvement after prone positioning was 49% [19–100%]. Responders and non-responders to PP did not show any significant difference in demographic characteristics, chronic disease or ventilatory treatment before the first session of PP and the proportion of male sex was the same in each group (Table 1). No significant difference was found in the duration of PP (Table 2).

**Table 2:** Clinical outcomes within the 28 days after inclusion

	Total population (*n* = 191)	Responders (*n* = 96)	Non-responders (*n* = 95)	*P*
PaO_2_/FiO_2_ response to prone positioning (%, mmHg)	49 [19–100]	100 [67–155]	19 [3–31]	< 0.001
Duration of prone positioning (hours)	16 [16–17]	16 [16–16.7]	16 [16–17]	0.757
Tracheostomy—no (%)	113 (59.2)	46 (47.9)	67 (70.5)	**0.008**
Duration of MV, days	18 [11–28]	18 [10–27]	18 [12–29]	0.432
Attempted extubation—no (%)	39 (20.4)	33 (34.3)	6 (6.3)	** < 0.001**
Weaning failure—reintubation no (%)	22 (18.8)	17 (17.7)	5 (5.3)	0.093
VAP—no (%)	105 (55)	53 (55.2)	52 (54.7)	0.885
Steroid use	133 (70%)	72 (75%)	61 (64%)	0.083
Non pulmonary infections—no (%)	72 (37.7)	37 (38.5)	35 (36.8)	0.882
Cardiovascular complications—no (%)	31 (16.2)	13 (13.5)	18 (18.9)	0.333
Digestive complications—no (%)	8 (4.2)	5 (5.2)	3 (3.2)	0.721
Neurologic complications—no (%)	17 (8.9)	9 (9.4)	8 (8.4)	1.000
Renal Replacement therapy—no (%)	43 (22.5)	22 (22.9)	21 (22.1)	1.000
Veno-venous ECMO—no (%)	3 (1.6)	0 (0.0)	3 (3.2)	0.121
ICU length of stay, days	22 [14–35]	22[15–35]	21[14–35]	0.994
VFD at 28 days, days	4.5 ± 7.1	6.3 ± 8.1	2.7 ± 5.6	** < 0.001**
ICU mortality—no (%)	83 (43.4)	32 (33.3)	51 (53.7)	**0.006**

Values are median (IQR) except for VFD (Mean ± SD). Significant *P* values are highlighted in bold

*FiO*_*2*_  inspired fraction of oxygen, *C*_*RS*_  respiratory system compliance measured in supine position, *PaO*_*2*_  arterial oxygen partial pressure, *MV*  mechanical ventilation, *NIV*  non-invasive ventilation, *CPAP*  continuous positive airway pressure, *HFNO*  high flow nasal oxygen, *VFD*  ventilator free days, *VAP*  ventilator associated pneumonia, *DP*  driving pressure

Globally, the lowest PaO_2_/FiO_2_ ratio observed during the first 5 days of ICU stay was 90 [69–113] and the two groups did not differ either for the lowest PaO_2_/FiO_2_ ratio or for the ARDS stage according to the Berlin definition [8]. Non-responders had significantly higher Pplat and lower C_RS_ during the first 5 days of ICU stay.

**Fig. 1:**
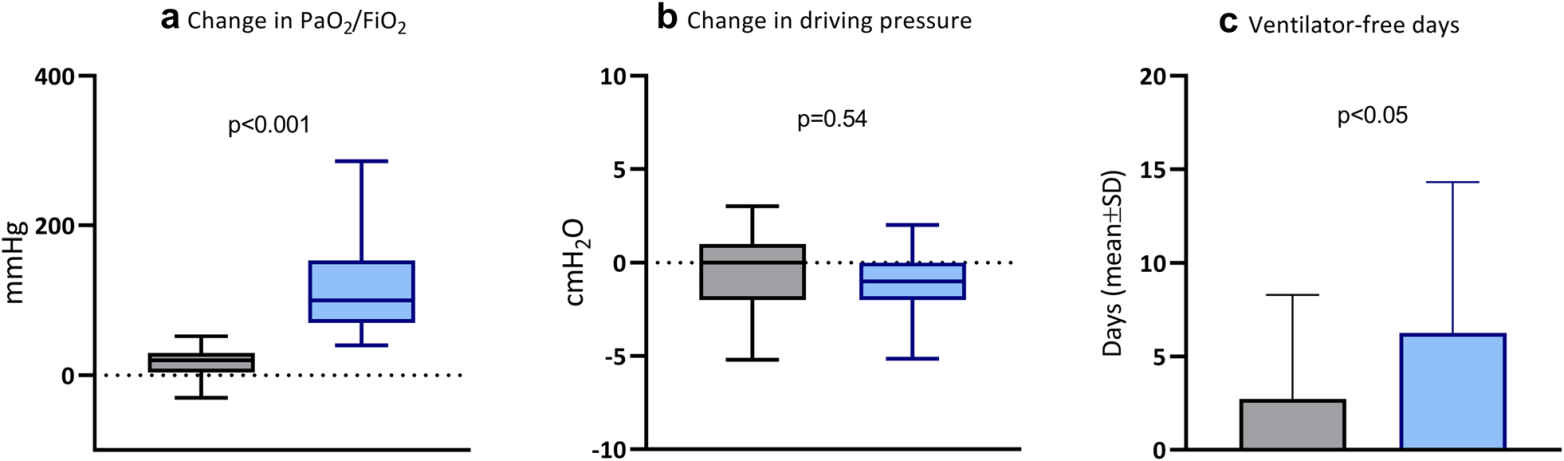
Physiologic effect of the first prone positioning session and impact on patient outcome. Effect of the first proning session on change in PaO_2_/FiO_2_ ratio (**a** PaO_2_/FiO_2_ in after prone position minus PaO_2_/FiO_2_ before prone position), change in driving pressure (**b** driving pressure after prone position minus driving pressure before prone position) and ventilator-free days (**c**) in responders (blue) and non-responders (grey) patients with acute respiratory distress syndrome related to COVID-19

Before PP, neither the PaO_2_/FiO_2_ ratio (responders: 101 [80–127] cmH_2_O, non-responders: (105 [90–130] cmH_2_O, *P* = 0.10) and DP (responders: 13 [10–16] cmH_2_O, non-responders: (14 [11–16] cmH_2_O, *P* = 0.16) were significantly different among groups. After the PP session, PaO_2_/FiO_2_ was 210 [161–276] in responders and 127 [100–150] mmHg (*P* < 0.001) in non-responders and the DP was slightly but significantly lower in responders as compared to non-responders (12 [10–14] vs 13 [11–15] cmH_2_O, *P* = 0.003). PFdiff was different between the two groups, as expected by study design, but DPdiff was not (Fig. 1a, b). Moreover, there was no significant correlation between PFdiff and DPdiff (*r* = − 0.06; *P* = 0.38, Additional file 2: Figure S2).

### Clinical outcomes in responders and non-responders

Clinical outcomes and complication rates during ICU stay in the global population and in the two groups are summarized in Table 2. Median duration of invasive mechanical ventilation and ICU length of stay were not significantly different between the groups. In responders, as compared to the non-responders group, the number of VFDs was significantly higher (mean ± SD 6.3 ± 8.1 vs 2.7 ± 5.6 days, *P* < 0.001) (Fig. 1c), the tracheostomy rate was lower (47.9% vs 70.5% *P* = 0.008) as well as the ICU mortality (33.3% vs 53.7%, *P* = 0.006). No differences in the cardiovascular, digestive, neurologic, infective and renal complications were found.

**Fig. 2:**
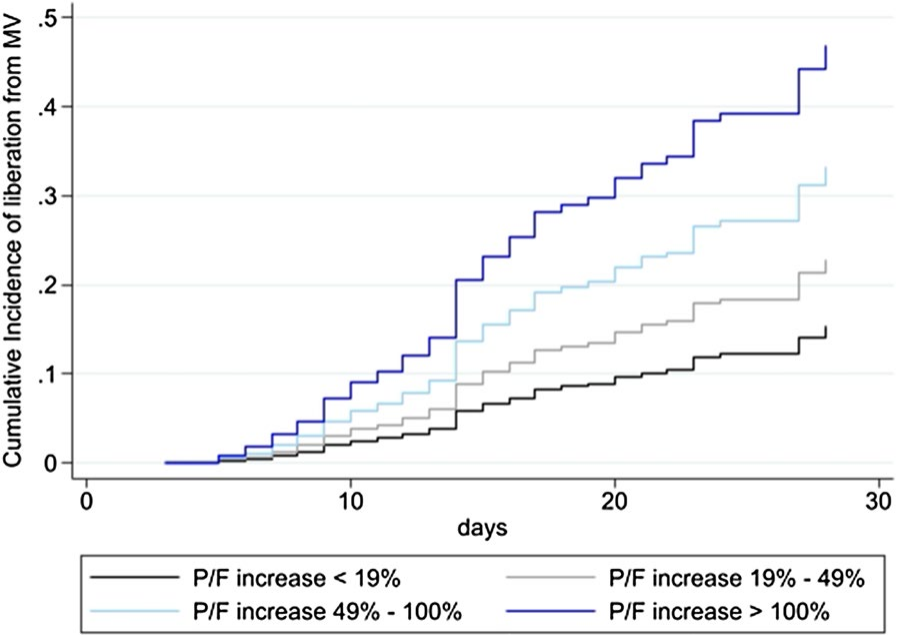
Cumulative incidence of liberation from mechanical ventilation. Cumulative incidence of liberation from mechanical ventilation (MV) over 28 days after intubation. The four curves represent the cumulative incidence functions related to the quartiles of PaO_2_/FiO_2_ response to prone positioning referred to the multivariate model (Table 3)

The competing risk regression analysis (Table 3) showed that an increase in PaO_2_/FiO_2_ after the first PP session was independently associated with a greater chance of liberation from mechanical ventilation at 28 days together with lower age, higher PaO_2_/FiO_2_ ratio during the first 5 days and the absence of renal, pulmonary, neurologic and cardiovascular complications. Specifically, in our population, for each quartile increase in terms of PaO_2_/FiO_2_ response the subhazard ratio for being free from invasive mechanical ventilation at 28 day increase of 1.563 (95% CI 1.329—1.838, *P* < 0.001, Fig. 2).

**Table 3:** Competing-risk regression analysis for liberation from mechanical ventilation with death as the competing event

Variable (reference level)	Univariate analysis			Multivariate analysis		
	SHR	95% CI	*P*	SHR	95% CI	*P*
Age	0.964	0.943–0.986	0.002	0.971	0.946–0.996	**0.025**
Sex (male)	0.679	0.305–1.511	0.342			–
BMI	1.011	0.959–1.065	0.689			–
SOFA score at admission	0.865	0.770–0.970	0.013			–
SAPS II score	0.970	0.937–1.004	0.076			–
Hypertension (yes)	0.700	0.507–0.967	0.031			–
Chronic ischemic heart disease (yes)	0.468	0.146–1.494	0.200			–
COPD (oxygen therapy/CPAP) (yes)	0.845	0.208–3.431	0.814			–
Chronic kidney disease (yes)	1.216	0.281–5.266	0.793			–
Diabetes (yes)	0.768	0.451–1.310	0.333			–
Chronic liver disease (MELD > 10)	1.054	0.295–3.765	0.936			–
Need for renal replacement therapy (yes)	0.208	0.093–0.466	0.001	0.244	0.113–0.526	** < 0.001**
Late onset VAP (yes)	0.290	0.174–0.483	< 0.001	0.280	0.174–0.450	** < 0.001**
C_RS_ < 40 ml/cmH_2_O in the first 5 days	1.021	0.990–1.053	0.195			–
Steroid use (yes)	1.860	0.852–4.060	0.119			–
PaO_2_/FiO_2_ variation after pronation^b^	1.370	1.187–1.582	< 0.001	1.563	1.329–1.838	** < 0.001**
Cardiovascular complications (yes)	0.180	0.0752–0.433	< 0.001	0.194	0.088–0.427	** < 0.001**
Neurologic complications (yes)	0.376	0.165–0.856	0.020	0.296	0.110–0.798	**0.016**
Digestive complications (yes)	0.267	0.046–1.583	0.146			–
Extra-pulmonary infections (yes)	0.698	0.486–1.000	0.050			–
DP before prone positioning	0.971	0.890–1.058	0.500			–
ARDS PaO_2_/FiO_2_ class (severe)^a^	1.775	1.143–2.755	0.011	1.738	1.116–2.705	**0.014**
Infection during ICU stay (yes)	0.698	0.486–1.000	0.050			–

Significant *P* values are highlighted in bold

*SHR* subdistribution hazard ratio (*SHR* 1 no association between the covariate and the corresponding cumulative incidence function, *SHR* > 1 an increase of the covariate value is associated with an increased risk of liberation from mechanical ventilation, *SHR* < 1 implies the opposite), *DP* driving pressure, *VAP* ventilator associated pneumonia, *C*_*RS*_ compliance of the respiratory system, *BMI* Body mass index, *COPD* chronic obstructive pulmonary disease, *CPAP* continuous positive airway pressure, *ICU* intensive care unit

^a^Per class points increase, class 1 = severe ARDS, class 2 = moderate ARDS. Reference level in branches for each covariate

^b^Per quartile variation, 1st quartile (< 19%), 2nd quartile (19–49%), 3rd quartile (49–100%), 4th quartile (> 100%)

## Discussion

In this secondary analysis of critically ill COVID-19 patients we analyzed if the sustained oxygenation improvement after the first PP session could be associated to ICU outcome in terms of time to liberation from mechanical ventilation, complication rates and mortality. We found that, in severe COVID-19-related ARDS, the sustained PaO_2_/FiO_2_ improvement after the first prone positioning was progressively related a to lower mechanical ventilation time and ICU mortality.

Severe COVID-19 is characterized by dyspnea, a respiratory rate of 30 or more breaths per minute, a blood oxygen saturation of 93% or less, a PaO_2_/FiO_2_ ratio of less than 300 mmHg, or infiltrates in more than 50% of the lung field within 24 to 48 h from the onset of symptoms [21]. PP has been rapidly adopted by intensivists once the first wave spread out to the ICUs worldwide, mainly for its positive effect on arterial oxygen content. This can be consequence of several mechanisms, which are largely dependent on the stage of the disease. Despite its sound physiological basis, PP determines a variable oxygenation response across COVID-19 patients, some improving dramatically oxygenation and others not. Recruitment of dorsal lung regions due to the lung edema shift from vertebral to sternal lung, which, furthermore, continued to receive most pulmonary blood flow (at least in non COVID-19-related ARDS), is the main mechanism thought to be involved in the oxygenation improvement during PP [22]. When this happens, *C*_RS_ improves, and DP decreases because of the wider surface available for ventilation.

When analyzing the oxygenation in COVID-19 patients returning to supine position after the first PP session, the variable persistence of oxygenation improvement was found both in noninvasively and invasively ventilated patients [7, 15, 16]. However, this finding has never been previously linked with patients’ outcome. We found that the sustained oxygenation improvement after the first PP session was independently associated with a reduced duration of mechanical ventilation and mortality rate.

In a previous study by Lee et al. [23] in non-COVID-19 ARDS, a sustained oxygenation after PP was associated to an improvement of respiratory system mechanics. In their paper, indeed, only responders increase *C*_RS_ after resupination, while non-responders did not. In our population, responders had a slightly lower DP after PP, but both responders and non-responders had a comparable decrease in DP—and presumably in lung recruitment—after PP. Since the improvement in DP was not different between responders and non-responders, it cannot explain per se why the oxygenation increased only in the responders group.

To support this, a recent work by Haddam et al. found that the gas exchange improvement after PP could not be predicted by the variation of dorsal aeration measured by lung ultrasound [24]. Therefore, several mechanisms, beside lung recruitment, are involved in the PaO_2_/FiO_2_ increase following PP in ARDS and this is probably even more true for COVID-19-related ARDS, where the vascular impairment can be responsible for a defective hypoxic pulmonary vasoconstriction [4]. A new CT scan study comparing COVID-19 ARDS to an historical non-COVID ARDS population, found, indeed, that for comparable lung aeration and compliance, COVID-19 ARDS has a significantly higher percentage of hypoxemia [25]. This confirms the hypothesis that COVID-19-related ARDS is a specific “vasocentric” phenotype of ARDS [26]. The oxygenation response to PP may, therefore, be a hint of at partially preserved ventilation/perfusion matching and, therefore, an indirect sign of disease extension. Patients not improving oxygenation after PP may, therefore, highlight an extended damage of both the alveolar and vascular structures. A recent observational study demonstrated that transesophageal echocardiography monitoring is feasible, sensitive and promising in tracking individual hemodynamic response to PP, which may be unpredictably deleterious in some patients [27]. The heterogeneous effect on the right ventricle output may, indeed, help to understand the different responsiveness to PP seen in these patients. Future studies are needed to address this key physiopathological point.

Previous studies in non-COVID ARDS found that an oxygenation improvement after PP was marginally [13] or not associated [14] with mortality. Despite mortality was not the primary outcome of this study, we believe that the association between response to PP and mortality may be a peculiar characteristic of COVID-19-related ARDS and that further studies need to specifically address this point.

Non-responders had a prolonged duration of mechanical ventilation, an increased risk of death and a higher rate of tracheostomy compared to responders. This was not unexpected, since a reduced response to PP was independently associated to a higher risk of prolonged liberation from mechanical ventilation, while no differences were found in the complication rates.

Since the oxygenation response to the first PP can highlight patients at major risk of death, it may be used to inform who may benefit from a further level of assistance. Beside PP, indeed, other interventions can be used to increase oxygenation in COVID-19, like inhaled nitric oxide [5, 28], intravenous Almitrine [29], ECMO [30]. The reduced oxygenation response to PP may be, therefore, helpful to select patients needing alternative ventilatory treatment. Indeed, the only three patients that in our population underwent ECMO were in the non-responders group.

A prolonged time of prone positioning (36 h) was recently suggested to help preserving the oxygenation improvement after resupination [31]. In our population, both responders and non-responders had the same time of PP, but it is worth to explore in future studies if non-responders may need a prolonged session of prone positioning to fully exploit the potential of the maneuver. We found that a sustained oxygenation improvement after prone positioning was associated with better outcome; whether this was linked to a higher organ oxygen delivery, to a different stage of the disease or to a different mechanism linked to PP disease must be explored by future studies. Poor response to prone positioning, moreover, could be potentially used to identify patients that are at higher risk of prolonged weaning and, therefore, modify the policy of tracheostomy, sedation and ventilation. During prone positioning, all patients were paralyzed and ventilated in volume-controlled ventilation. Recent evidence [32] show that spontaneous breathing could be beneficial during prone position and the effect of spontaneous breathing during prone positioning in COVID-19 patients has to be explored. Moreover, despite no differences were found in driving pressure change, PEEP and recruitment may have played a role in some patients. Further studies are needed to assess the impact of PEEP [33], lung recruitment and/or recruitability [34, 35] on PP response.

Our study has several limitations. First, the ventilatory treatment and weaning were not standardized among participating, thus adding potential confounding factors. Second, for many variables, we asked the participating centers to collect the lowest values within the first 5 days of ICU stay, thus possibly missing important data on the precise time course of these variables. Third, several experimental COVID-19 therapies were tested in different centers during the conduction of present study. Forth, we did not evaluate thrombosis among complications, since this parameter can be difficult to be assessed, both for micro and macro thrombosis. Finally, we analyzed the response to the first prone positioning session. Further studies should evaluate if the response to subsequent PP sessions could be useful in predicting outcome. In our analysis, we grouped patients based on the oxygenation response to PP. Before performing the maneuver, no single variable was predictive of the response. Moreover, all patients started PP per clinical decision, and therefore, it is possible to assume that the severity of patients in the groups was the same and this was confirmed by the baseline characteristics of the groups. The only mechanical difference among the two groups in the first 5 days of ICU stay was the lowest Crs, since this was slightly higher in responders. Despite this, in both groups PP was decided on oxygenation and not on respiratory mechanics. Post-hoc power analysis revealed that the primary outcome (VFDs) had a power of 0.92, meaning that there is an 92% chance of detecting a difference as statistically significant, if in fact a true difference exists.

## Conclusions

A sustained oxygenation response after the first PP session in COVID-19 ARDS patients is an independent predictor of prolonged liberation from mechanical ventilation and ICU survival. Oxygenation improvement to PP is not related to improvement in DP. Further studies are needed to evaluate if the oxygenation response to PP can be used in the decision-making process in severe mechanically ventilated COVID-19 patients.

## Supplementary Information

**Additional file 1: Fig. S1.** Flow chart of data analysis.

**Additional file 2: Fig. S2.** Correlation between PaO2/FiO2 difference (PFdiff, after minus before prone position) and driving pressure difference (DPdiff, after minus before prone position). Pearson R correlation in the entire population.

**Additional file 3.** ICU-RER collaborators list.

## Data Availability

On reasonable request.

## Abbreviations

ARDS: Acute respiratory distress syndrome
COVID-19: Coronavirus 19 disease
CPAP: Continuous positive airway pressure
Crs: Respiratory system compliance
DP: Driving pressure
DPdiff: Driving pressure difference
ECMO: Extra-corporeal membrane oxygenation
FiO_2_: Inspired oxygen fraction
HFNO: High flow nasal cannula
ICU: Intensive care unit
IQR: Interquartile range
NIV: Non-invasive ventilation
PaO_2_: Partial arterial oxygen content
PEEP: Positive end-expiratory pressure
PFdiff: P/F difference
PP: Prone positioning
Pplat: Plateau pressure
SAPS: Simplified acute physiology score
SD: Standard deviation
SOFA: Sequential organ failure assessment
VA⁄Q ˙: Ventilation/perfusion
VFD: Ventilatory free days
