# Supplementary figures and images for: Sustained oxygenation improvement after first prone positioning is associated with liberation from mechanical ventilation and mortality in critically ill COVID-19 patients: a cohort study
Source: Ann Intensive Care. 2021 Apr 26;11:63. doi: 10.1186/s13613-021-00853-1 (PMC8072095; doi:10.1186/s13613-021-00853-1)

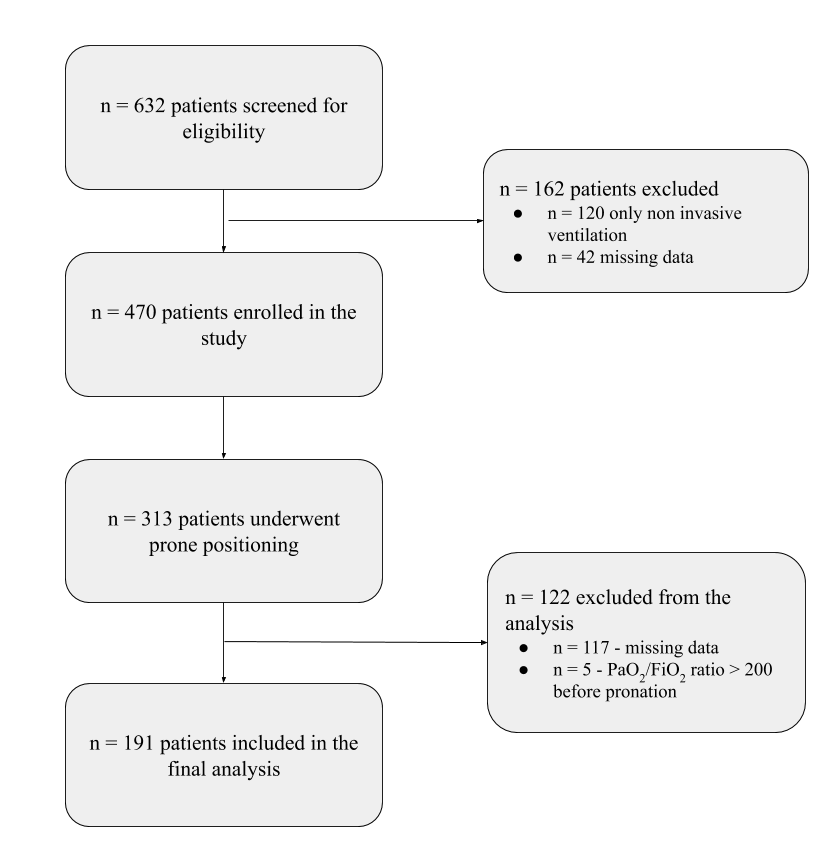

Supplement: Supplementary file 1 — Additional file 1: Fig. S1. Flow chart of data analysis. [file 13613_2021_853_MOESM1_ESM.png]

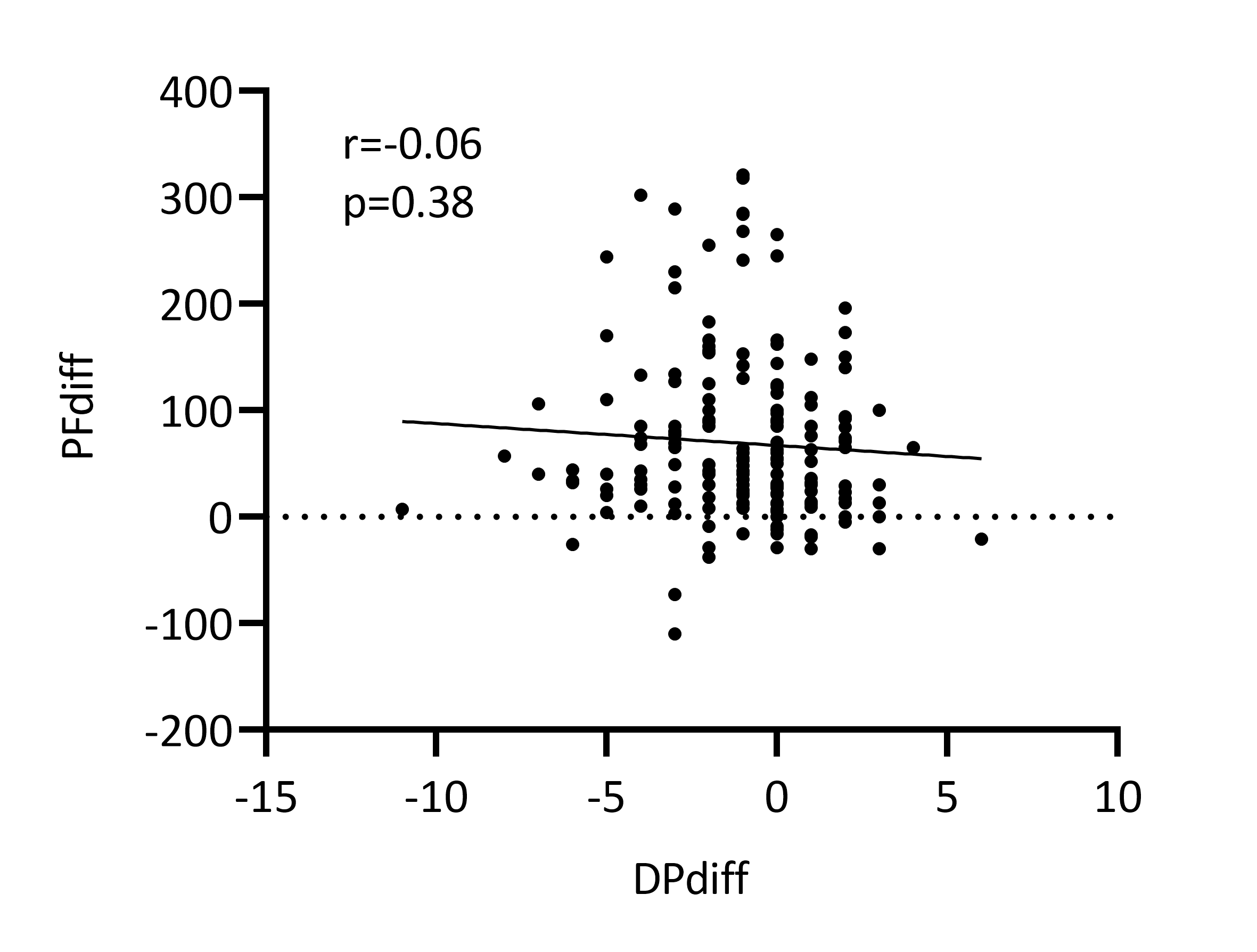

Supplement: Supplementary file 2 — Additional file 2: Fig. S2. Correlation between PaO2/FiO2 difference (PFdiff, after minus before prone position) and driving pressure difference (DPdiff, after minus before prone position). Pearson R correlation in the entire population. [file 13613_2021_853_MOESM2_ESM.tif]
