# Supplementary material for: Sustained oxygenation improvement after first prone positioning is associated with liberation from mechanical ventilation and mortality in critically ill COVID-19 patients: a cohort study
Source: Ann Intensive Care. 2021 Apr 26;11:63. doi: 10.1186/s13613-021-00853-1 (PMC8072095; doi:10.1186/s13613-021-00853-1)
Supplement: Supplementary file 3 — Additional file 3. ICU-RER collaborators list. [file 13613_2021_853_MOESM3_ESM.docx]

**ICU-RER COVID-19 Collaboration – List of collaborators (to be indexed and searchable into PubMed)**

- *Maggiore Hospital Carlo Alberto Pizzardi, Bologna, IT*: Marco Tartaglione, Valentina Chiarini, Virginia Buldini, Carlo Coniglio, Federico Moro, Clara Barbalace, Mario Citino.
- *Bellaria Hospital, Bologna, IT*: Nicola Cilloni, Lorenzo Giuntoli, Angela Bellocchio, Emanuele Matteo.
- *Sant’Orsola-Malpighi University Hospital, Bologna, IT*: Giacinto Pizzilli, Antonio Siniscalchi, Chiara Tartivita, Francesco Matteo.
- *Imola Hospital, Bologna, IT*: Annalisa Marchio, Igor Bacchilega.
- *Infermi Hospital, Rimini, IT*: Laura Bernabé, Sonia Guarino, Elena Mosconi.
- *M.Bufalini Hospital, Cesena, IT*: Luca Bissoni, Lorenzo Viola.
- *Santa Maria Annunziata Hospital, Firenze, IT*: Tommaso Meconi, Vittorio Pavoni.
- *SS. Trinità Hospital, ASL Novara, IT*: Aline Pagni, Patrizia Pompa Cleta, Marco Cavagnino.
- *Bentivoglio Hospital, Bentivoglio, IT*: Anna Malfatto, Angelina Adduci, Silvia Pareschi.
- *University Hospital of Modena, Modena, IT*: Gabriele Melegari, Jessica Maccieri, Elisa Marinangeli.
- *Azienda Ospedaliera SS. Antonio e Biagio e Cesare Arrigo, Alessandria, IT*: Fabrizio Racca.
- *University of Ferrara, Azienda Ospedaliero-Universitaria S. Anna, Cona, Ferrara, IT*: Marco Verri, Giulia Falò, Elisabetta Marangoni.
- *Villa Erbosa Hospital, San Donato Group, Bologna, IT*: Francesco Boni.
- *Santa Maria delle Croci Hospital, Ravenna, IT*: Giulia Felloni, Federico Domenico Baccarini.
- *Morgagni-Pierantoni Hospital, Forlì, IT*: Marina Terzitta, Stefano Maitan.
- *Azienda USL Toscana Centro, Prato, IT*: Filippo Becherucci, Maddalena Parise, Francesca Masoni

**Radiology Collaborators (to be indexed and searchable into PubMed)**

- *Maggiore Hospital Carlo Alberto Pizzardi, Bologna, IT*: Michele Imbriani, Paolo Orlandi, Francesco Monetti
- *Bellaria Hospital, Bologna, IT*: Giorgia Dalpiaz
- *Sant’Orsola-Malpighi University Hospital, Bologna, IT*: Rita Golfieri, Federica Ciccarese
- *Imola Hospital, Bologna, IT*: Antonio Poerio
- *Infermi Hospital, Rimini, IT*: Francesco Muratore, Fabio Ferrari
- *M.Bufalini Hospital, Cesena, IT*: Martina Mughetti
- *SS. Trinità Hospital, ASL Novara, IT*: Loredana Franchini, Ersenad Neziri
- *Bentivoglio Hospital, Bentivoglio, IT*: Marco Miceli
- *Santa Maria delle Croci Hospital, Ravenna, IT*: Maria Teresa Minguzzi, Lorenzo Mellini
- *Morgagni-Pierantoni Hospital, Forlì, IT*: Sara Piciucchi
- *Azienda USL Toscana Centro, Prato, IT*: Maurizio Bartolucci
